# Irrigation level and substrate type on the acclimatization and development of mandacaru (*Cereus jamacaru* DC.): an emblematic cactus from Brazilian semiarid region

**DOI:** 10.1038/s41598-023-47929-5

**Published:** 2023-11-23

**Authors:** Carlos Alberto Lins Cassimiro, Juliane Maciel Henschel, Vanessa Gabrielle Nóbrega Gomes, Rita de Cássia Alves, Pollyana Karla da Silva, Emmanuel Moreira Pereira, Mônica Tejo Cavalcanti, Diego Silva Batista, Fabiane Rabelo da Costa Batista

**Affiliations:** 1 Graduate Program in Agricultural Sciences (Agroecology), Federal University of Paraíba, Bananeiras, Paraíba 58220-000 Brazil; 2 National Institute of the Semiarid, Av. Francisco Lopes de Almeida, s/n, Serrotão, Campina Grande, Paraíba 58434-700 Brazil; 3 Graduate Program in Agronomy, Federal University of Paraíba, Areia, Paraíba 58397-000 Brazil; 4 Department of Agriculture, Federal University of Paraíba, Campus Universitário III, s/n, Bananeiras, PB 58220-000 Brazil

**Keywords:** Plant development, Plant physiology, Plant stress responses

## Abstract

Mandacaru is a cactus with great socioeconomic potential, but lack of information about its cultivation hinders its domestication. Here, we aimed to evaluate the acclimatization and vegetative development of mandacaru under different substrates and irrigation levels. For this, seeds inoculated in vitro were grown for 120 days, being transplanted to pots containing four types of substrate (S1—caatinga soil + gravel; S2—washed sand + organic matter + soil + charcoal; S3—washed sand + cattle manure + soil + sand; S4—commercial organic substrate). Pots were irrigated with 100% of the field capacity (FC) once-a-week, or with 50% FC twice-a-week, and kept in a greenhouse for six months. The experimental design was completely randomized, in a 4 × 2 factorial scheme, with six replications. Plant height and diameter, axial and radial growth rate, fresh and dry mass of stem and root, water content, and photosynthetic pigments were determined. Growth was affected mainly by the substrate, with S4 resulting in higher growth and pigment content, while S1 was impaired and S2 and S3 resulted in intermediate growth. The use of S4 and 100% FC once per week was the best condition for mandacaru.

## Introduction

*Cereus jamacaru* DC., popularly known as *mandacaru*, *cardeiro*, *mandacaru-de-boi*, and *mandacaru-de-faixo*, is a columnar cactus of the genus *Cereus*, which comprises 34 species, of which 10 are native and 8 endemic to Brazil^1^. This is a perennial species with cylindrical, succulent, semi-woody stems that reach up to 12 m in height and 25–60 cm in diameter, with white flowers and edible fruits^2,3^. In Brazil, this species is distributed across the Caatinga, Cerrado, and Atlantic Forest biomes, with higher occurrence in the arid and semi-arid regions, being of fundamental ecological importance for the fauna and populations during periods of water scarcity^1,4^. In this context, mandacaru has a great socioeconomical importance in the Brazilian Semi-arid region, being used as a source of wood, animal and human feed, medicine, handicraft, ornamentation, cellulose, powdered biosorbents, etc^3,5–7^.

Despite the great socioeconomic potential, the domestication of the mandacaru is still incipient, and these products are obtained mainly through extractive exploitation, which represents a threat to the conservation of this species. The slow growth and lack of information on management for seedling production are major obstacles to the domestication of this species^2^. In this sense, the development of techniques that optimize the propagation and growth of mandacaru are imperative to the conservation and domestication of this species. In vitro cacti cultivation is a propagation technique that uses seeds or plant tissues, under aseptic and controlled climatic conditions, favoring healthy and homogeneous seedling development and allowing mass production of seedlings^8,9^. In cacti, this technique has been related to an increase in the survival rate, resulting in more vigorous plants, which can contribute to the preservation, regeneration and conservation ex situ and in situ^10,11^.

The acclimatization is a critical step after in vitro propagation, as the seedlings face environmental conditions completely different from those where they developed initially^12^. In addition to the light and temperature, the type of substrate and irrigation level are crucial to allow plant survival and development^13,14^. Cacti from semi-arid regions, such as the Brazilian Caatinga, are commonly found in rocky, sandy areas with little organic matter^15^. Substrates such as natural soil, sand, volcano rocks, anthill soil, goat, cattle or poultry manure, rice husks, and charcoal, have been pointed as suitable for other cacti cultivation^14,16^. These substrates differ widely in their chemical and physical composition, with different mineral composition, water-holding capacity, aeration and porosity, making it necessary to determine which substrate is the best option for each species. Similarly, it is imperative to determine the optimal level and frequency of irrigation in species such as mandacaru, because most studies on cactaceous irrigation have been conducted on the forage cacti *Opuntia* spp^13,17^.

Considering the great socioeconomic potential and the intense extractive exploitation of the mandacaru, as well as the difficulties in propagation and domestication of this species, and the enormous lack of information about the management of its cultivation, it is essential to establish techniques that favor the conservation and commercial production of this species. Here, we hypothesize that the use of different substrates and irrigation levels can improve the development and acclimatization of mandacaru. In this context, this study aimed to evaluate the acclimatization and vegetative development of mandacaru plants under different types of substrates and irrigation levels.

## Results

### Growth and development of mandacaru

**Figure 1:**
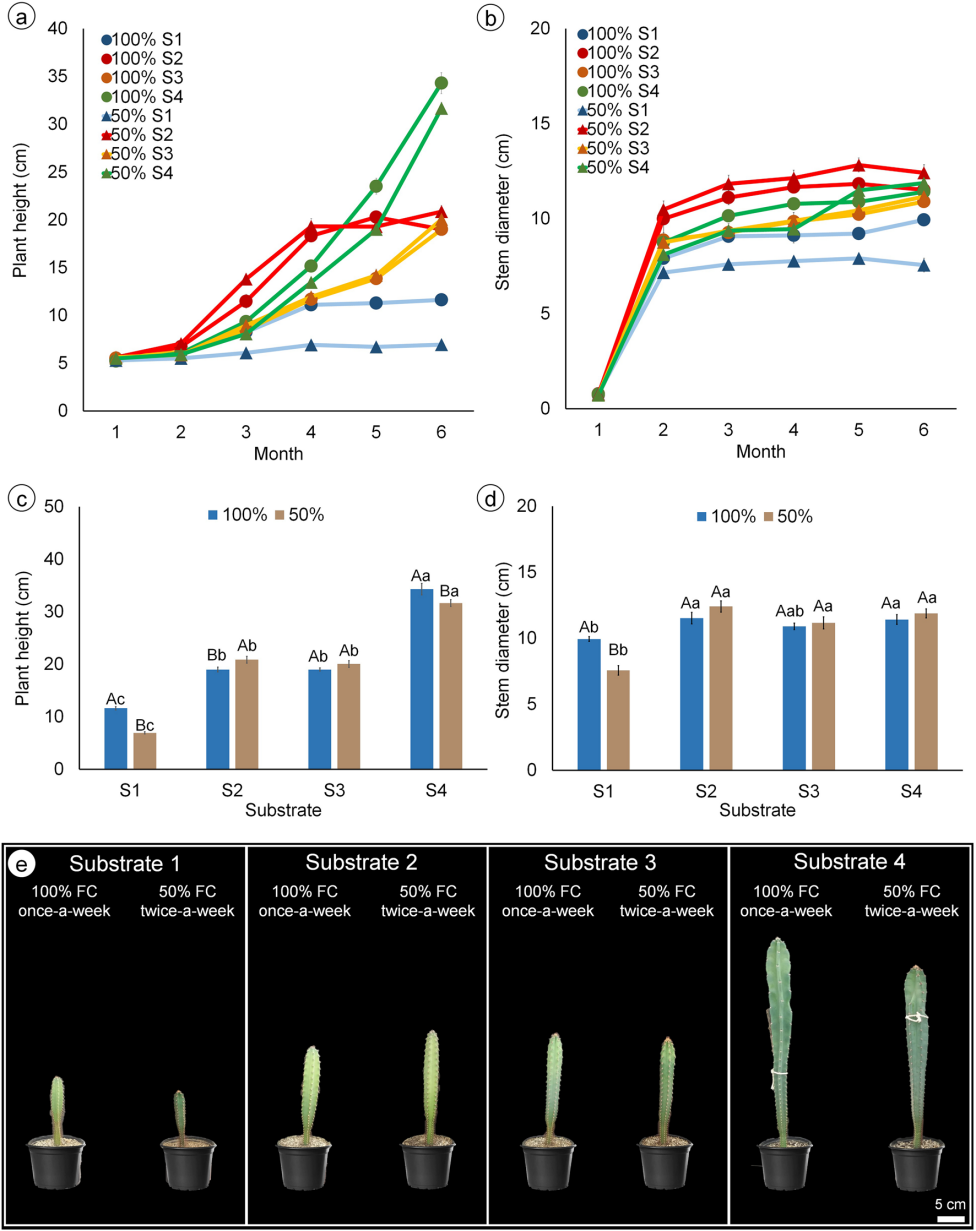
Plant height and stem diameter of mandacaru (*Cereus jamacaru*) plants during (**a,b**) and at the end of the 6 months of acclimatization in a greenhouse (c, d, and e). Plants were grown in four different substrates (S1: caatinga soil + gravel; S2: washed sand + organic matter + soil + charcoal; S3: washed sand + cattle manure + soil + sand; S4: commercial organic substrate) and irrigated either with 100% FC once-a-week, or with 50% FC twice-a-week. Bars represent the mean of six plants ± standard error. Capital and lowercase letters indicate differences between irrigation levels and among substrates by Tukey’s test (*P* ≤ 0.05), respectively.

The initial developmental stages of mandacaru were not affected by the type of substrate and irrigation level, however, differences in height and stem diameter began to be observed from the third month of acclimatization, with significant differences in the sixth month (Fig. 1a, b). At the end of the sixth month, there was a significant interaction between the types of substrates and irrigation levels, being the type of substrate, the main factor affecting the development of mandacaru, with the commercial substrate (S4) resulting in the highest growth and the caatinga soil (S1) in the lowest (Fig. 1a–e). Irrigation level, in turn, slightly affected the growth of mandacaru, with 50% FC reducing plant height of plants grown with S1 and S4, and increasing that of S2, compared to 100% FC (Fig. 1c). Stem diameter was lower in S1 plants, being reduced by 50% FC, compared to 100% FC, while it was not affected by irrigation level in the other substrates (Fig. 1d). The combination between S1 and 50% FC provided twice-a-week resulted in the lowest plant height and stem diameter.

**Figure 2:**
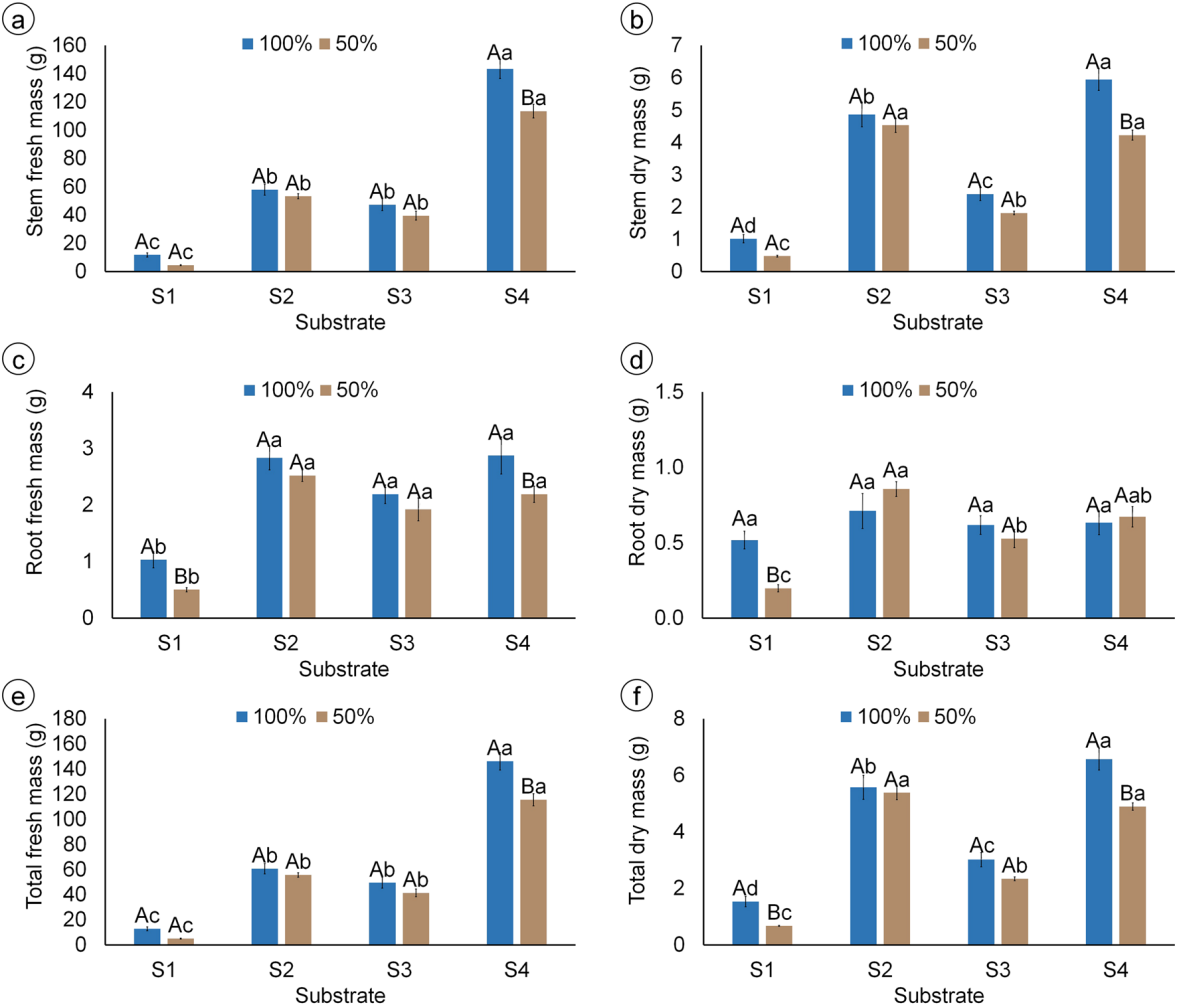
Biomass production of mandacaru (*Cereus jamacaru*) plants after 6 months of acclimatization in a greenhouse. Plants were grown in four different substrates (S1: caatinga soil + gravel; S2: washed sand + organic matter + soil + charcoal; S3: washed sand + cattle manure + soil + sand; S4: commercial organic substrate) and irrigated either with 100% FC once-a-week, or with 50% FC twice-a-week. Bars represent the mean of six plants ± standard error. Capital and lowercase letters indicate differences between irrigation levels and among substrates by Tukey’s test (*P* ≤ 0.05), respectively.

Like the plant height and diameter, S4 also resulted in the highest stem fresh and dry mass, while S2 and S3 had intermediary and S1 had the lowest stem biomass (Fig. 2a, b). The stem fresh mass of 100% FC plants with S4 was 12-fold higher than with S1, while in 50% FC, plants with S4 had stem fresh mass 25-fold higher than with S1 (Fig. 2a). The stem dry mass of plants irrigated with 100% FC and grown with S2 was higher than S3, and lower than S4, while under 50% FC it was higher than in S3 but did not differ from S4 (Fig. 2b). The irrigation level only affected the stem biomass of plants grown with S4, with 50% FC reducing stem fresh and dry mass compared to 100% FC (Fig. 2a, b). Total fresh and dry mass showed the same pattern as stem mass, with highest total biomass with S4 and lowest with S1, and 50% FC reducing the fresh mass in S4, and the dry mass in S1 and S4, compared to 100% FC (Fig. 2e, f).

At both irrigation levels, root fresh mass did not differ between substrates S2, S3, and S4, while S1 resulted in the lowest values (Fig. 2c). Root dry mass, in turn, did not differ among substrates when irrigated with 100% FC, but was lower in S1 and S3 irrigated with 50% FC, compared to S2 and S4 (Fig. 2d). Moreover, the irrigation level of 50% FC reduced root fresh mass in S1 and S4, and root dry mass in S1, while not affecting the root growth in other substrates (Fig. 2c, d).

**Figure 3:**
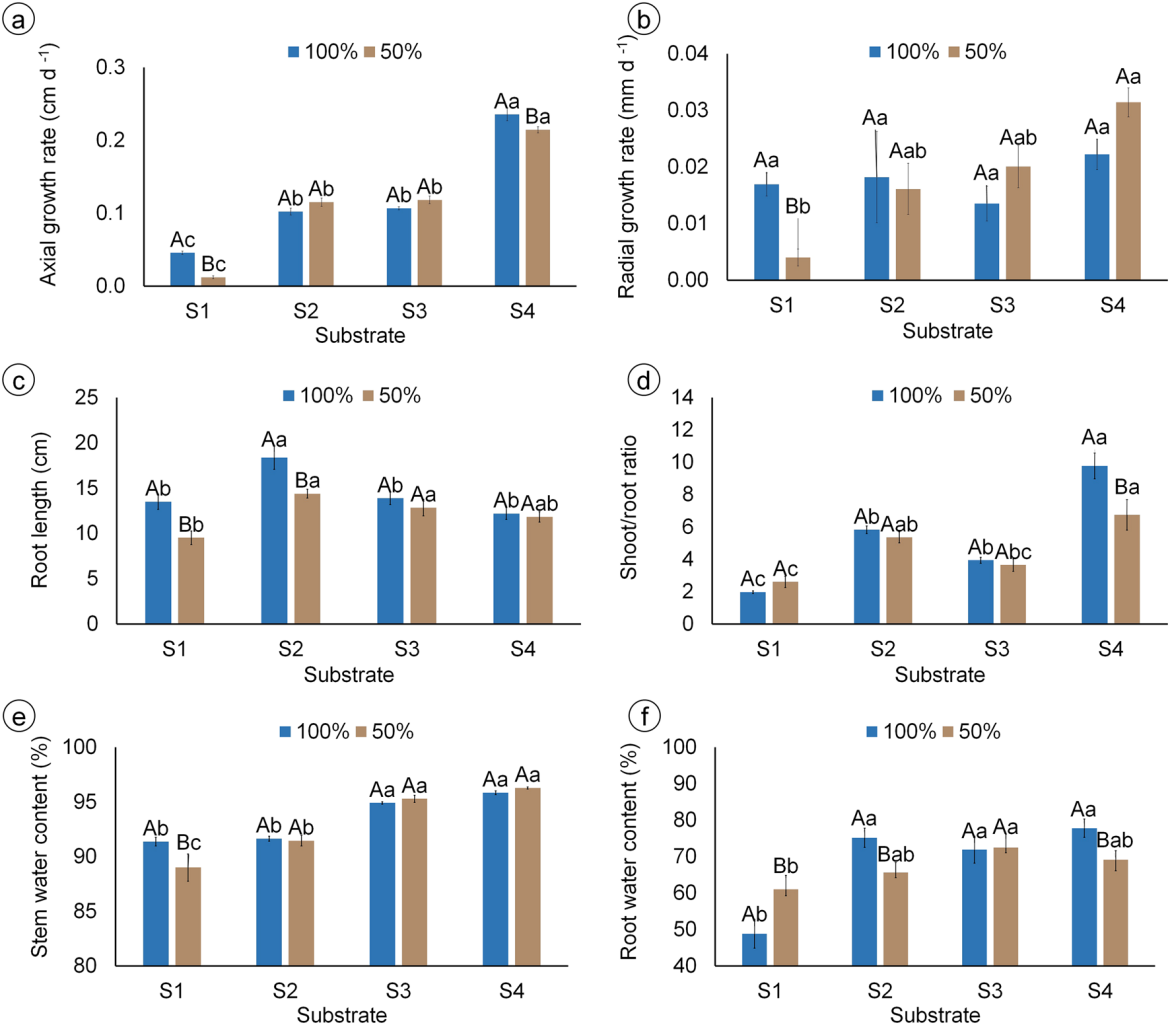
Growth parameters and water content of mandacaru (*Cereus jamacaru*) plants after 6 months of acclimatization in a greenhouse. Plants were grown in four different substrates (S1: caatinga soil + gravel; S2: washed sand + organic matter + soil + charcoal; S3: washed sand + cattle manure + soil + sand; S4: commercial organic substrate) and irrigated either with 100% FC once-a-week, or with 50% FC twice-a-week. Bars represent the mean of six plants ± standard error. Capital and lowercase letters indicate differences between irrigation levels and among substrates by Tukey’s test (*P* ≤ 0.05), respectively.

According to the biomass production, the axial growth rate was higher in S4 and lower in S1, with irrigation of 50% FC reducing these rates (Fig. 3a). Radial growth rate, in turn, did not differ among substrates when pots were irrigated with 100% FC, while it was lower in S1 under 50% FC, compared to 100% FC and to the other substrates (Fig. 3b). Plants grown with S2 and 100% FC had the longest roots, while those grown in S1 and 50% FC irrigation had the shortest roots (Fig. 3c). Shoot/root ratio showed that plants grown in S4 allocated more biomass to stems instead of roots compared to the other substrates, which was reduced by irrigation with 50% FC compared to 100% FC (Fig. 3d). Plants grown with S1, in turn, allocated less biomass to shoots in relation to roots, independently of irrigation (Fig. 3d).

Stem water content was higher in plants grown with S3 and S4 under both irrigation levels, while plants in S1 and irrigated with 50% FC had the lowest stem water content (Fig. 3e). Root water content, in turn, did not differ among substrates S2, S3, and S4, being lower in S1 in both irrigation levels (Fig. 3f). Moreover, 50% FC reduced root water content in S2 and S4, but increased in S1, when compared to 100% FC.

### Photosynthetic pigments content

**Figure 4:**
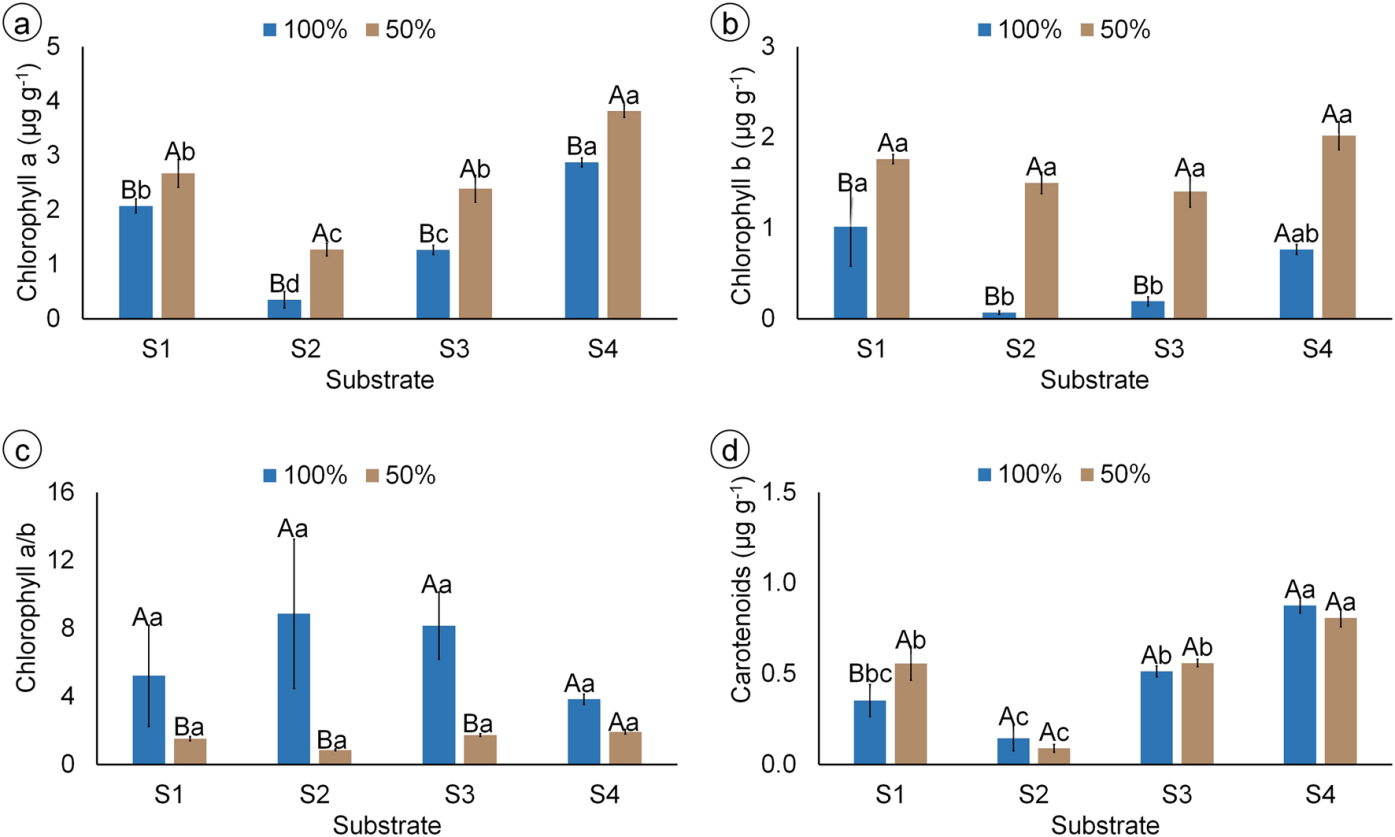
Photosynthetic pigments contents of mandacaru (*Cereus jamacaru*) plants after 6 months of acclimatization in a greenhouse. Plants were grown in four different substrates (S1: caatinga soil + gravel; S2: washed sand + organic matter + soil + charcoal; S3: washed sand + cattle manure + soil + sand; S4: commercial organic substrate) and irrigated either with 100% FC once-a-week, or with 50% FC twice-a-week. Bars represent the mean of six plants ± standard error. Capital and lowercase letters indicate differences between irrigation levels and among substrates by Tukey’s test (*P* ≤ 0.05), respectively.

The highest chlorophyll *a* content was obtained in plants grown in S4 irrigated with 50% FC, while the highest chlorophyll *b* content was obtained with 50% FC, regardless of the substrate used (Fig. 4a, b). These differences led to higher chlorophyll *a*/*b* ratios in plants irrigated with 100% FC (Fig. 4c). The highest total carotenoids content was obtained in S4, and the lowest was found in S2, regardless of the irrigation (Fig. 4d).

### Grouping of substrates and irrigation levels

**Figure 5:**
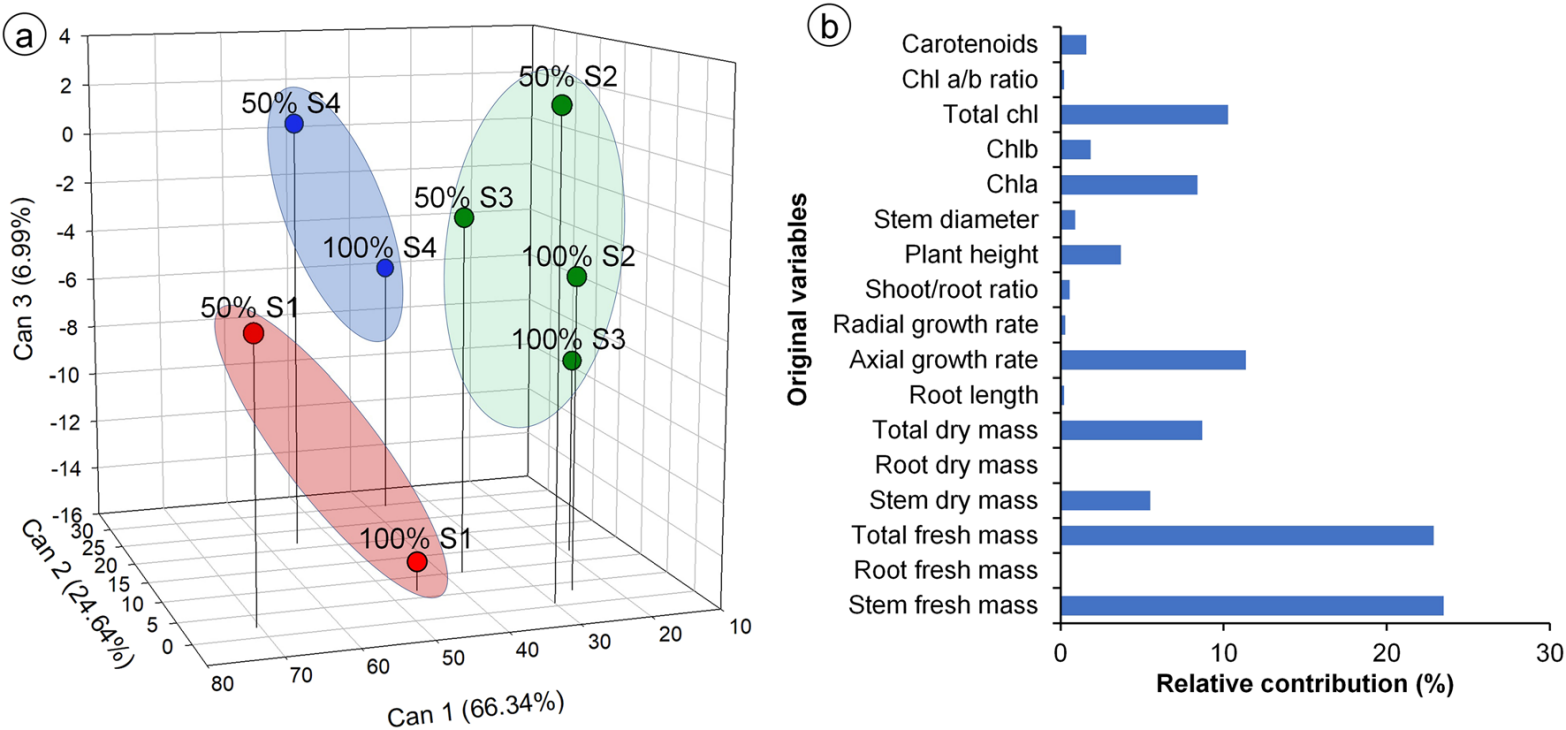
Canonical variables obtained from original variables, in mandacaru (*Cereus jamacaru*) plants grown in four different substrates (S1: caatinga soil + gravel; S2: washed sand + organic matter + soil + charcoal; S3: washed sand + cattle manure + soil + sand; S4: commercial organic substrate) and irrigated either with 100% FC once-a-week, or with 50% FC twice-a-week. (**a**) Three-dimensional scatter plot of first three canonical variables (% total variance explained by each canonical component is indicated in parentheses; treatments indicated by the same color were assembled into the same group by the Tocher optimization method and the generalized squared interpoint distance of Mahalanobis); (**b**) relative contributions of original variables, calculated using the Singh method, to the canonical variables.

The first three canonical variables were attributable to > 97.9% of variability between treatments, allowing for a three-dimensional scatterplot representation (Fig. 5a). Moreover, the four original variables that most contributed to the total variance were the stem fresh mass (23.5%), total fresh mass (22.9%), axial growth rate (11.4%), and total chlorophylls (10.3%), respectively (Fig. 5b). The Singh method allowed the separation of treatments into three groups: group 1 (red circles) comprised the plants grown with caatinga soil, S1, under both irrigation levels (100% S1 and 50% S1); group 2 (blue circles) comprised the commercial substrate S4 under both irrigation levels (100% S4 and 50% S4); group 3 (green circles) comprised the plants grown with substrates S2 and S3 under both irrigation levels (100% S2, 50% S2, 100% S3, and 50% S3) (Fig. 5a).

## Discussion

Suitable substrates must have physical and chemical characteristics that provide good conditions for plant growth, such as good porosity to improve drainage and aeration to avoid diseases, adequate nutrient availability, ensuring good nutrition without causing salt stress^13,16^. In this study, the commercial substrate (S4) resulted in the best vegetative development for mandacaru, evidenced by greater plant height, stem diameter, biomass accumulation and axial and radial growth rate. This can be attributed to the characteristics of this substrate, which has good aeration and drainage, resulting in less compaction, as well as high availability of macro and micronutrients and organic matter in its composition. In contrast, the substrate containing Caatinga soil and gravel (S1), had low water retention and organic matter, with high compaction, which resulted in less aeration and water availability. Consequently, S1 resulted in lower development of mandacaru plants, regardless of the irrigation level. In addition, the root fresh mass was reduced in plants grown with S1, which may be a consequence of the high compaction and low availability of water and nutrients in this substrate, also limiting the stem growth.

Considering that an adequate water balance is required for the formation of turgor pressure, which is necessary for cell expansion and division^18,19^, the reduction in mandacaru growth may have occurred due to decreased water availability and water balance in S1. In fact, S1 resulted in lower water content in stems and roots, further corroborating that this substrate may have caused water deficit to plants. In this context, it might be thought that substrates with higher water retention would increase plant growth, however, the substrates S2 and S3 that required higher water volume to saturate (140 mL and 110 mL, respectively), resulted in intermediary growth, being higher than in S1 but lower than in S4 (both saturated with 50 mL). Water excess causes plant stress, leading to stomatal closure, chlorophyll degradation, leaf senescence and reduced photosynthesis, also reducing oxygen availability and respiration, and resulting in oxidative stress^20^. Thus, the growth of mandacaru in S2 and S3 suggest that these substrates may have resulted in water excess to plants. The use of organic inputs, such as cattle manure, has been pointed as essential to the cultivation of cacti^21,22^. However, the substrate containing cattle manure used here (S3) resulted in lower mandacaru growth compared to the commercial substrate (S4), indicating that organic matter and nutrients are not the mainly factor affecting mandacaru growth, but possibly also other factors like the physical properties of the soil.

In contrast, under water deficit conditions, plants increase water uptake by allocating more biomass to the roots than to the aboveground^23^. In fact, plants under low water availability (S1) had greater biomass allocated to roots, while plants with higher water availability (S4) allocated more biomass to stems. Despite the lower shoot/root ratio and root fresh mass in S1, the root length did not differ between S1 and S4. Furthermore, roots were shorter in S1 irrigated with 50% twice-a-week, compared to 100% once-a-week. Since the shallow root system in cacti is a mechanism to limit water loss to dry soils and maximize water uptake after watering, optimizing the use of limited and intermittent rainfall^24^, this may help explain the shorter roots in the substrate with higher compaction, which received less water twice a week.

In general, the lowest chlorophylls and carotenoids contents were found in S2, which had the more yellowish aspect of plants. S4 plants, in turn, had a more greening aspect and resulted in the highest pigments contents. Chlorophyll *a* is the most abundant photosynthetic pigment and is directly involved in photosynthesis as a component of the reaction centers of photosystems, being that its increase raises photosynthetic rates, consequently affecting plant development^25^. In this context, as S4 resulted in the highest chlorophyll *a* content, this suggests plants in this substrate had better photosynthetic performance, which reflected in the higher growth. Intriguingly, the lowest chlorophyll *a* content was observed in the substrates with intermediary growth (S2 and S3), and not in the substrate with the lowest growth (S1). As water excess induce chlorophyll degradation^20,26^, this may help to explain the lower chlorophyll *a* and *b* content in S2 and S3, as these substrates received the higher amounts of water. It is noteworthy that 50% FC, provided twice-a-week, increased chlorophyll *a* and *b* in all substrates.

Chlorophyll *b*, in its turn, is a pigment found in the light harvesting complex of photosystems, being involved in photoprotection of the photosynthetic apparatus and in the absorbance of wavelengths complementary to those of chlorophyll *a*^27^. In this sense, the increased chlorophyll *b* found in S1, and in all substrates under 50% FC, might represent a photoprotection mechanism, indicating that these conditions might have caused stress to plants. This may also be related to the reduced chlorophyll a/b ratio, as it is an indicator of photosynthetic efficiency^28,29^. Although the increase in carotenoid levels has been related to defense responses against stresses^30,31^, here, substrates S1, S2, and S3, which impaired mandacaru growth, showed the lowest carotenoid contents. By contrast, the S4 had the highest carotenoid content, suggesting that this substrate provided better conditions for the development of mandacaru plants, including the synthesis of pigments.

Stem fresh mass was the original variable that most contributed to the separation among treatments into the three groups, with groups being formed according to the lowest (S1), highest (S4), and intermediary growth of stems (S2 and S3). On the other hand, there was no separation between irrigation levels within each substrate, indicating that the type of substrate was the main factor affecting mandacaru growth. In addition, chlorophylls contents also contributed to the separation of groups, with the higher contents being found in group 2 (S4). In the context of ornamental production of mandacaru, S4 was the best substrate because it resulted in higher chlorophyll content and faster growth of mandacaru, but under natural conditions, excessive growth in height may result in lodging of the plant, low density per area and, therefore, greater susceptibility to herbivore attacks. In this context, although the caatinga soil has resulted in lower growth of mandacaru, it would be a good option for seedling production aiming the recovery of degraded areas, because it may be related to greater survival under natural conditions. As the irrigation with 100% FC, provided only once a week, resulted in better development of mandacaru, this watering regime was the most indicated, also resulting in lower maintenance during cultivation. Another aspect observed in this study, was the fast growth of mandacaru in S4, reaching an average height of 30 cm. Other studies reported that, when propagated by sowing the seeds directly on the substrate, mandacaru plants take about 5 months to reach 19 cm, and 12 months to reach 25 cm^2,32^. In sum, this study points that the in vitro seed propagation, combined with S4 and 100% FC during acclimatization, are the ideal conditions to produce mandacaru for ornamental and productive purposes.

## Methods

### Plant material and experimental location

Mandacaru (*Cereus jamacaru* DC. ssp. *jamacaru*) seeds were obtained from mature fruits collected of different individual plants occurring in the legal reserve forest area at National Institute of the Semi-Arid (INSA), located in the municipality of Campina Grande, Paraíba, Brazil (7°16′49″ S, 35°58′33″ W, and 508 m altitude). Voucher specimens of *Cereus jamacaru* DC. ssp. *jamacaru* were deposited in the Herbarium Jayme Coelho de Moraes at the Center for Agricultural Sciences, Federal University of Paraíba, Areia, Paraíba, Brazil (EAN-22157). We germinated the seeds in vitro and grown for 120 days in the in vitro Plant Cultivation Laboratory (INSA), then, transferred seedlings to a greenhouse covered with plastic film (150 µm) and 50% black shade net, with average temperature of 31 °C and relative humidity of 46%.

### In vitro propagation and acclimatization

We surface sterilized seeds by washing with 70% ethanol (v/v) for 1 min and, then by dipping for 15 min in a sodium hypochlorite solution (2.5%) containing the surfactant Tween 20^®^ (0.03%). Next, we washed the seeds three times in deionized and autoclaved water and placed in filter paper for drying, and inoculated in glass flasks containing autoclaved MS medium^33^, with sucrose (30 g L^−1^), inositol (100 mg L^−1^), and 0.6% agar, pH 5.8. Seeds were sterilized and inoculated in a laminar flow hood. Afterward, we transferred the flasks to a growth room at 25 ± 2 °C, with 16 h/8 h photoperiod, and light intensity of 47 µmol m^2^ s^−1^. After 120 days, the seedlings were removed from the flasks, washed with water for 1 min, and standardized by size (6 ± 1 cm in height). Then, we transplanted the seedlings into pots with different substrates and irrigation levels and transferred them to a greenhouse covered with plastic film (150 µm) and 50% black shade net, with average temperature of 31 °C and relative humidity of 46%.

### Substrates and irrigation levels

A total of 48 40-day-old seedlings were transferred to pots with 90 mL of capacity containing four different substrates and grown in the greenhouse where was monitored for 6 months. The composition and chemical attributes of the substrates are shown in Table 1.

**Table 1:** Chemical attributes and description of the substrates used in this study.

Substrate	pH	Ca^2+^ (cmol_c_ kg^−1^)	Mg^2+^(cmol_c_ kg^−1^)	K^+^(cmol_c_ kg^−1^)	Na^+^ (cmol_c_ kg^−1^)	H + Al (mg kg^−1^)	P (g kg^−1^)	TC (g kg^−1^)	TN (g kg^−1^)	TS (g kg^−1^)
Substrate 1 (S1)	6.48	13.24	0.32	0.61	1.05	5.56	85.83	6.63	1.19	0.17
Substrate 2 (S2)	6.14	17.32	3.47	2.34	1.61	15.13	204.58	137.20	3.31	0.80
Substrate 3 (S3)	7.05	12.41	2.55	2.55	1.07	7.48	193.64	85.12	4.84	1.02
Substrate 4 (S4)	6.26	15.01	3.40	1.69	1.49	16.45	218.46	151.80	4.06	0.07

Substrate	Composition	Water volume to saturate (100% FC) (mL)								
Substrate 1 (S1)	Caatinga soil + gravel (1:1)	50								
Substrate 2 (S2)	Washed sand + organic matter + soil + charcoal (1:1:1:1)	140								
Substrate 3 (S3)	Washed sand + cattle manure + soil + sand (1:1:1:1)	110								
Substrate 4 (S4)	Commercial organic substrate (Terraplant^®^)	50								

*TC* total carbon, *TN* total nitrogen, *TS* total sulfur, *FC* field capacity.

Plants were irrigated either with 100% of the field capacity (FC), provided once-a-week, or with 50% of FC, applied twice-a-week. The field capacity of each substrate was determined by the lysimetric drainage method. For this, 150 mL of water were slowly added to 100 g of each substrate, and the volume of the drained solutions were measured 24 h later. Then, field capacity was determined by the difference between the volume of water applied (150 mL) and the volume that was drained. The difference corresponded to the water retained on the substrate (100% FC). 50% FC corresponded to the half volume applied on 100% FC treatment.

### Growth analysis

The height and the diameter of the stem were measured monthly, with ruler and pachymeter, respectively. The stem height was used to calculate axial growth rate, and the stem diameter was used to calculate radial growth rate^34^. At the end of the experiment (6 months of acclimatization), plants were collected and separated in stems and roots to determine the organ fresh mass, root length, and photosynthetic pigments. Stem, root and total dry mass were determined after drying the plants at 65  ± 0.5 °C in an oven with forced air circulation, until reaching constant weight. The water content of the stem and root was determined by the difference between fresh and dry mass and expressed in percentages.

### Photosynthetic pigments content

The content of chlorophyll *a*, chlorophyll *b*, and carotenoids was determined according to^35^, with modifications. For this, discs with 250 mg were collected from stems, placed into Eppendorf tubes containing 2 mL of 80% acetone (v/v), and kept in the dark, at 4 °C, for 48 h. After this, the extract was read in a spectrophotometer at 663 nm, 647 nm, and 470 nm.

### Experimental design and statistical analysis

The experimental design was completely randomized, in a 4 × 2 factorial scheme (substrates × irrigation levels), with six replicates, and each experimental unit corresponding to one pot with one plant. Data were tested for normality and homogeneity using the Shapiro–Wilk and Bartlett tests, respectively. Statistically significant differences were determined by analysis of variance (ANOVA; F test) and means of the significant variables were compared by Tukey’s test (*P* ≤ 0.05) using the Genes software^36^. The distance between treatments was identified by canonical discriminant analysis using three-dimensional scatter plots. Groups were identified using Tocher optimization and generalized Mahalanobis square interpostal distance (D2). Grouping quality was assessed using the co-optic correlation coefficient (r). The relative contribution of each variable to the discrimination of treatments was quantified by Singh's criterion^37^.

### Research involving plants

All procedures were conducted in accordance to the institutional, national, and international guidelines and legislation. All the permissions and licenses, following institutional, national, and international guidelines and legislation, were obtained to collect the plants.

## Conclusions

The use of the commercial organic substrate, together with irrigation at 100% of the field capacity provided once a week, was the best condition for the cultivation of mandacaru (*Cereus jamacaru*), since it resulted in larger plants, with greater accumulation of biomass and chlorophylls, also reducing the management during cultivation. Our findings contribute to the scientific knowledge on the topic, since is the first to indicate the best substrate and irrigation levels for mandacaru. Also, considering the lack of information on the acclimatization and cultivation, will support future studies and cultivation of this species. From an ecological and conservationist point of view, despite the Caatinga soil resulted in lower growth, it can be a good option when aiming the reintroduction of the species in their natural environment. Despite the challenges of studying soil conditions due to the plethora of biotic and abiotic effects that interact complexly, future studies can advance by defining factors such as tolerance to drought and high temperatures.

## Data Availability

The datasets generated during and/or analyzed during the current study are available from the corresponding author on reasonable request.
